# Supplementary material for: Aggregating soft labels from crowd annotations improves uncertainty estimation under distribution shift
Source: PLoS One. 2025 Jun 9;20(6):e0323064. doi: 10.1371/journal.pone.0323064 (PMC12148080; doi:10.1371/journal.pone.0323064)
Supplement: S1 Text — The first task we consider is recognizing textual entailment (RTE). In the RTE task, a model must predict whether a hypothesis is entailed (i.e. supported) by a given premise. For training, we use the Pascal RTE-1 dataset [19] with crowd-sourced labels from [20]. The dataset consists of 800 premise-hypothesis pairs annotated by 164 different non-expert annotators with 10 annotations per pair. The inter-annotator agreement (IAA) is 0.629 (Fleiss κ). As an out-of-domain test set, we use the Stanford Natural Langauge Inference dataset (SNLI) [21], where we transform the task into binary classification by collapsing the “neutral” and “contradiction” classes into a single class. Distribution shift in this case comes in the form of data distribution (source data: news and various NLP datasets from pre-2005; target: crowd-sourced sentences). (PDF) [file pone.0323064.s001.pdf]

Supporting information

Full Dataset Descriptions

Recognizing Textual Entailment (RTE) The first task we consider is recognizing textual entailment (RTE). In the RTE task, a model must predict whether a hypothesis is entailed (i.e. supported) by a given premise. For training, we use the Pascal RTE-1 dataset [19] with crowd-sourced labels from [20]. The dataset consists of 800 premise-hypothesis pairs annotated by 164 different non-expert annotators with 10 annotations per pair. The inter-annotator agreement (IAA) is 0.629 (Fleiss κ). As an out-of-domain test set, we use the Stanford Natural Langauge Inference dataset (SNLI) [21], where we transform the task into binary classification by collapsing the “neutral” and “contradiction” classes into a single class. Distribution shift in this case comes in the form of data distribution (source data: news and various NLP datasets from pre-2005; target: crowd-sourced sentences).

Part-of-Speech Tagging (POS) The POS tagging task is a sequence tagging task, where the goal is to predict the correct part-of-speech for each token in a sentence. For training data, we use the Gimpel dataset from [22] with the crowd-sourced labels provided by [23] mapped to the universal POS tag set in [24]. The dataset consists of 1000 tweets (17,503 tokens) labeled with Universal POS tags and annotated by 177 annotators. Each token received at least 5 annotations. The IAA is 0.725 and the average annotator accuracy with respect to the gold labels is 67.81%. We use the publicly available sample of the Penn Treebank POS dataset [25] accessed from NLTK [26] as our out-of-domain test set, which consists of 3,914 sentences from Wall Street Journal articles (100,676 tokens). Distribution shift on this task is based on the data distribution (source: tweets, target: news).

Toxicity Detection To measure performance on a highly subjective task, we use the toxicity detection dataset created as a part of the Google Jigsaw unintended bias in toxicity classification competition. The dataset we use comes from [27], which annotated 25,500 comments from the original Civil Comments dataset. The pool of annotators is specifically selected and split into multiple rating pools based on self-indicated identity group membership. As this is a highly subjective task, the IAA in terms of Krippendorff's α is 0.196. We randomly split the dataset into training and test, and for the test data we use the annotations in the original crowd-sourcing task; in other words, using a completely separate annotator pool that isn't selected based on identity groups. The source of distribution shift for this task comes from the annotator pools (i.e., the data is the same but the annotators change; source: unrestricted annotator pool, target only self-identifying African American and LGBTQ annotators):

Image Classification The training dataset for image classification comes from the 10,000 images in the CIFAR10 test set re-annotated by crowd workers on Amazon Mecahnical Turk (AMT) [2]. The final dataset contains 511,400 annotations, with 51 annotations on average per image, and an IAA of 0.91 Krippendorff's α. For the test data, we use the CINIC10 dataset [28], which contains 210,000 images from Imagenet rescaled down to 32x32 to match the CIFAR10 image size. Here, distribution shift comes from the data distribution (source: CIFAR10 images, target: scaled down Imagenet images).

Significance Tests

We show a more conservative test of significance, applying the Bonferroni correction across the number of tests (N = 56) in Figure 13 – Figure 16. The differences from the less conservative significance test are: for POS tagging, no method is statistically significantly different for raw performance, and the only significance in uncertainty estimation are aggregation being statistically significantly better than Softmax, and WaWA being statistically significantly better than DS. On toxicity detection, for raw performance, softmax is no longer statistically significantly better than WaWA and ZBS, and for uncertainty estimation, standard is no longer statistically significantly worse than DS, GLAD, and MACE, softmax is no longer statistically significantly better than DS, GLAD, and MACE, and ZBS is no longer statistically significantly worse than Aggregation. For RTE and image classification there is no change.

Fig 13. Significance testing for the RTE task. We apply the Bonferroni correction across the total tests (N = 56). Green indicates the method in the row is significantly better than the method in the column. Red indicates the method in the row is significantly worse than the method in the column. Grey indicates no statistically significant difference.

Fig 14. Significance testing for the POS task. We apply the Bonferroni correction across the total tests (N = 56). Green indicates the method in the row is significantly better than the method in the column. Red indicates the method in the row is significantly worse than the method in the column. Grey indicates no statistically significant difference.

Fig 15. Significance testing for the Toxicity task. We apply the Bonferroni correction across the total tests (N = 56). Green indicates the method in the row is significantly better than the method in the column. Red indicates the method in the row is significantly worse than the method in the column. Grey indicates no statistically significant difference.

Evaluation Metrics

F1 We used the sklearn implementation of precision_recall_fscore _support for F1 score, which can be found here: `https://scikit-learn.org/stable/modules/generated/sklearn.metrics.precision_recall_fscore_support.html`. Briefly:

$$p = \frac{tp}{tp + fp}$$

$$r = \frac{tp}{tp + fn}$$

$$F1 = \frac{2 * p * r}{p + r}$$

where tp are true positives, fp are false positives, and fn are false negatives.

Fig 16. Significance testing for the Image Cls. task. We apply the Bonferroni correction across the total tests (N = 56). Green indicates the method in the row is significantly better than the method in the column. Red indicates the method in the row is significantly worse than the method in the column. Grey indicates no statistically significant difference.

Calibrated Log-Likelihood The calibrated log-likelihood is defined in [30] as a method to fairly compare uncertainty estimation between models on the same test set. The key observation is that in order to obtain a fair comparison, one must first perform temperature scaling at the optimal temperature on the classifier output for each model under comparison. Additionally, this temperature must be optimized on an in-domain validation set. The procedure to calculate the calibrated log-likelihood is:

1. Split the **test set** in half, one half for validation and one half for test.
2. Optimize a temperature parameter T to minimize the average negative log-likelihood $-\frac{1}{n}\sum_i \log \tilde{p}(y_i = y_i^*|x_i)$, where $\tilde{p}_i = \text{softmax}(\frac{l_i}{T})$ and l_i is the logits of the classifier, on the validation half of the test set.
3. Measure the temperature scaled log-likelihood on the test half of the test set.

Following the suggestion from [30], we run this procedure 5 times on different splits of the test set and take the average test-half log-likelihood as the result.

Reproducibility

All NLP experiments were run using the RoBERTa base model released in the HuggingFace hub (`roberta-base`, `https://huggingface.co/roberta-base`) which has 125M parameters. All experiments with CIFAR10H/CINIC10 were run using a vision transformer with 85.8M parameters (HuggingFace: `google/vit-base-patch16-224-in21k`). We ran our experiments on a single NVIDIA TITAN RTX with 24GB of RAM.

Hyperparameters We found that performing a hyperparameter search using the in-domain validation set for hyperparameter selection can actually hurt our final performance – we tested this by performing a hyperparameter search for the RTE dataset, finding a general drop in both F1 performance and CLL on the out of domain test set, while failing to reduce the variance. The search was performed across the following values using a Bayesian hyperparameter search for 400 steps per setting:

- Learning rate: [1e-5, 1e-3]
- Batch size: {2, 4, 8, 16, 32, 64}
- Epochs: {1, 2, 3, 4, 5, 8, 10, 15}
- Warmup steps: {0, 20, 100, 200, 500, 1000}

As such, we use good hyperparameter settings for the class of base models used (RoBERTa), which have been shown to produce highly performant classifiers across many tasks [17]. We used a learning rate of 2e-5 with triangular learning rate schedule using 200 warmup steps. Models are trained for 5 epochs, using the best validation F1 for the final model. The average runtimes are: 50m00s (Toxicity), 70m00s (Image Classification), 2m28s (POS), 2m39s (RTE).
